# Does breastfeeding have a long-term positive effect on dental occlusion?

**DOI:** 10.4317/jced.56312

**Published:** 2019-10-01

**Authors:** Montserrat Boronat-Catalá, Carlos Bellot-Arcís, José-María Montiel-Company, José-Manuel Almerich-Silla, Montserrat Catalá-Pizarro

**Affiliations:** 1 Post-Graduated in Orthodontics, Department of Stomatology, Faculty of Medicine and Dentistry, University of Valencia, Valencia, Spain; 2 Assistant Lecturer, Orthodontics Teaching Unit, Department of Stomatology, Faculty of Medicine and Dentistry, University of Valencia, Valencia, Spain; 3 Associate Lecturer, Preventive Dentistry Teaching Unit, Department of Stomatology, Faculty of Medicine and Dentistry, University of Valencia, Valencia, Spain; 4 Tenured Lecturer, Preventive Dentistry Teaching Unit, Department of Stomatology, Faculty of Medicine and Dentistry, University of Valencia, Valencia, Spain; 5 Tenured Lecturer, Pediatric Dentistry Teaching Unit, Department of Stomatology, Faculty of Medicine and Dentistry, University of Valencia, Valencia, Spain

## Abstract

**Background:**

Breastfeeding produces beneficial effects on a baby’s early growth and development, and general well-being. Some studies have associated breastfeeding with better occlusal development. The aim of this study was to assess the long-term effect of breastfeeding on occlusal development in children.

**Material and Methods:**

A retrospective cohort study was conducted to evaluate the occlusions of 320 children belonging to the Spanish INMA Project cohort, monitored from gestation onwards. The association between the duration of breastfeeding and different occlusal traits in mixed dentition (overjet, overbite, openbite, midline displacement, incisor crowding, incisor spacing, diastema, maximum maxillary and mandibular irregularity, anterior and posterior crossbite) at the age of 9 was assessed, as well as the orthodontic treatment need as determined by the “Index of Orthodontic Treatment Need” and the “Dental Aesthetic Index”.

**Results:**

A statistically significant association between the duration of breastfeeding and posterior crossbite was found. The Odds Ratio for posterior crossbite was 2.52 (IC 95% 1.34-4.74) for children breastfed up to 16 weeks, 0.56 (IC 95% 0.29-1.08) for children breastfed 16 to 45 weeks, and for more than 45 weeks of breastfeeding it was 0.50 (IC 95% 0.19-1.32). No association was found between breastfeeding and the other examined occlusal traits, nor with the orthodontic treatment need.

**Conclusions:**

Breastfeeding for less than 4 months increases the risk of posterior crossbite. However, breastfeeding duration is not linked to other malocclusion traits nor is it linked to the orthodontic treatment need of nine-year-old children.

** Key words:**Breastfeeding, occlusion, malocclusion, dental development.

## Introduction

The WHO recommends exclusive breastfeeding during the first six months of life, as this reduces the risk of infectious diseases of the gastrointestinal tract and the respiratory system. In addition to the nutritional, immunological and psychological benefits for the baby, breastfeeding can also promote a better development of orofacial structures (1).

Breastfeeding can be seen as a natural orthopaedic appliance that promotes good craniofacial development, as the movements produced by the tongue and mandible during the suction of breast milk stimulate a better maxillary and mandibular growth (2). Furthermore, for the child to obtain milk during breastfeeding the stimulation of tongue and peribuccal muscles is required, whereas less effort is required for bottle-fed children to obtain milk, therefore causing less stimulation of the orofacial structures (3). For these reasons, breastfeeding could promote better occlusal development in primary dentition and the correct growth of the orofacial structures, and this effect could extend through into the mixed dentition stage.

The aim of this study is to investigate the influence of breastfeeding on the occlusion, which is a subject of current debate in the scientific literature.

## Material and Methods

This is a cross-sectional study in which, as part of broader research, a buccodental examination was carried out on boys and girls aged 9 belonging to the Spanish INMA Project cohort in Valencia (Spain) (4), in order to evaluate the occlusal traits and orthodontic treatment needs as determined by two internationally accepted and recognised epidemiologic indexes (5,6), Index of Orthodontic Treatment Need – IOTN (7) and Dental Aesthetic Index – DAI (8), and to determine any associations with the duration of breastfeeding. The cohort was recruited during the pregnant women’s first prenatal appointment at 10 - 13 weeks of gestation at La Fe Hospital in Valencia, Spain.

The study was approved by the Human Research Ethics Committee of the University of Valencia (H1372162226937). Prior to the dental examination, all the parents of the study participants completed and signed an informed consent form.

The children were examined clinically in a fully equipped dental chair at the University of Valencia Dental Clinic by only one dental examiner who had previously been calibrated by an orthodontic specialist for the use of the DAI and IOTN indexes, and the evaluation of occlusal traits using a >0.8 kappa value. Moreover, the intra- and inter- examiner reproducibility was greater than 0.80.

The dental examinations were carried out using an OMS-type periodontal probe and a No. 5 plain mouth mirror.

All the data required for the calculation of the DAI and IOTN was collected in a specific form. Dental examinations were carried out between November 2013 and May 2014.

The studied variables were as follows: overjet, overbite, openbite, midline displacement, incisor crowding, incisor spacing, diastema, maximum maxillary and mandibular irregularity, anterior crossbite, posterior crossbite and the orthodontic treatment need determined by the DAI and IOTN indexes. According to the IOTN index, grades 4 and 5 determine the need for orthodontic treatment and according to the DAI index, a score of ≥31 determines the need for orthodontic treatment.

Breastfeeding data was obtained from the INMA Project databases, which had been prospectively recorded during the perinatal period.

Statistical analysis was performed with SPSS. V22.0® software. The Chi-square test was employed for the comparison of proportions. The Student ‘t’ test and Analysis of Variance (ANOVA) were employed for the comparison of means. The significance level was established at *p*<0.05.

## Results

The study population was comprised of 320 individuals, 51.6% boys (165) and 48.4% girls (155), with an average age of 9.20 years (ranging from 8.26 to 10.22 years). The average age of the boys was 9.18 years, while that of the girls was 9.21 years. The average number of breastfeeding weeks was 24.7 weeks (IC 95% 22.6-26.8) and the range oscillated between 0 and 63 weeks.

To evaluate the association between breastfeeding duration in weeks, occlusal traits and orthodontic treatment need, the participants were divided into 3 groups: Group 1 (up to 16 weeks of breastfeeding), Group 2 (16 - 45 weeks of breastfeeding) and Group 3 (more than 45 weeks of breastfeeding).

The sample was distributed as follows: 36.6% (117) in Group 1, 41.6% (133) in Group 2 and 21.9% (70) in Group 3.  Table 1 and  Table 2 present the results regarding the association between breastfeeding duration in weeks and the different occlusal traits.

**Table 1:**
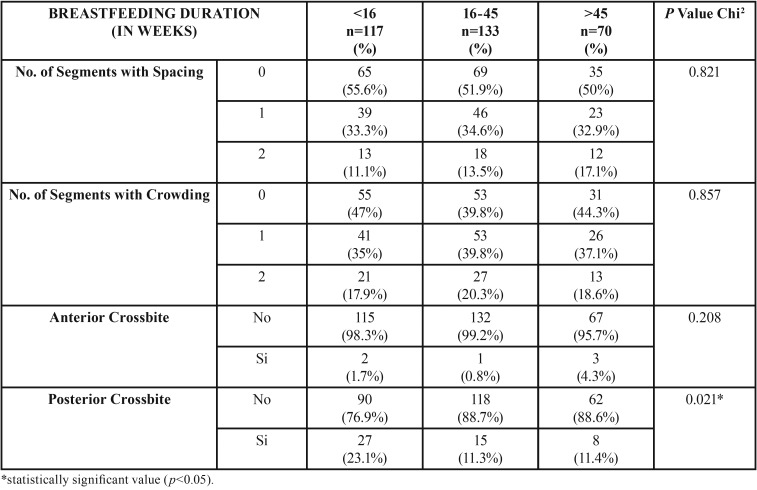
Association between number of weeks of breastfeeding and occlusal traits (1).

**Table 2:**
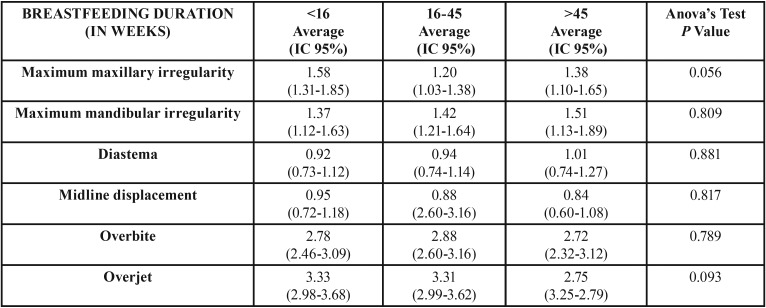
Association between number of weeks of breastfeeding and occlusal traits (2).

With the application of the Chi-square test only one statistically significant association was observed between breastfeeding duration and posterior crossbite, (*P* value = 0.021); it was observed that the infants breastfed for less than 16 weeks had a posterior crossbite prevalence of 23% while in infants breastfed between 16 to 45 weeks or more, the prevalence decreased by 11% ( Table 1). The average number of breastfeeding weeks for children with no posterior crossbite was 25.7 weeks (IC 95% 23.3-28.0), whereas the average for those with posterior crossbite was 19.3 weeks.

The Odds Ratio of crossbite in children breastfed for less than 16 weeks was 2.52 (IC 95% 1.339-4.743), so the risk of crossbite was 2.5 times greater for the children breastfed between 0 and 16 weeks than for those breastfed more than 16 weeks. The Odds Ratio of crossbite in children breastfed between 16 and 45 weeks rose to 0.56 (IC 95% 0.29-1.08) and 0.50 (0.19-1.32) in children breastfed for more than 45 weeks compared to those breastfed for less than 16 weeks.

 Table 3 shows the association between the groups established according to the length of breastfeeding in weeks and the orthodontic treatment need determined by the DAI and IOTN. A statistically significant association with either of those two indexes was not found.

**Table 3:**
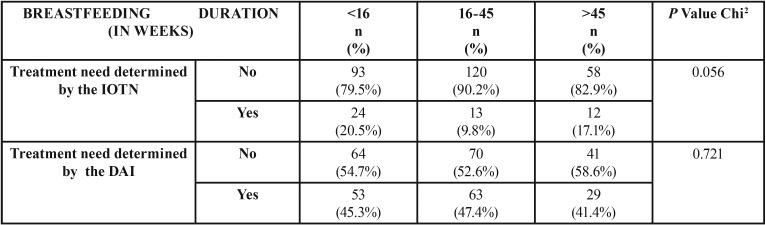
Summary of the number of weeks of breastfeeding in comparison with the orthodontic treatment need as determined by the IOTN and DAI.

## Discussion

Our findings agree with another study (9) that found an association between absence of breastfeeding or a short breastfeeding period with a greater prevalence of posterior crossbite in mixed dentition. Some authors also observed an association between breastfeeding and posterior crossbite in temporary dentition (3,10-17). Only one study found no association between breastfeeding duration and posterior crossbite (18). The results of the present study confirm the protective effect of breastfeeding against posterior crossbite, which extends through into mixed dentition. In this study the Odds Ratio of posterior crossbite in children breastfed for less than 16 weeks was 2.52; this means that the risk of developing a posterior crossbite in mixed dentition is 2.5 times higher in children breastfed for less than 16 weeks. These results also coincide with those of two systematic reviews (19,20) and a meta-analysis (21); both found that breastfeeding for longer periods of time is a protective factor against posterior crossbite.

The present study found no association between the duration of breastfeeding and the rest of the examined occlusal traits (overjet, overbite, openbite, midline displacement, incisor crowding, incisor spacing, diastema, maximum maxillary and mandibular irregularity, anterior crossbite and posterior crossbite). However, previous research found that anterior crossbite was more prevalent in children who had been breastfed for a short period (1,12,17,18,22-25). The results of the Dogramaci meta-analysis (26) also found a greater risk of developing an anterior openbite in shorter breastfeeding periods. However, the study by Sum (27), as in our own study, found no association between the duration of breastfeeding and overbite or openbite.

The study by Lescano de Ferrer *et al.* (11) found a lower prevalence of anterior crossbite in children who had been breastfed, although the present study found no association between these variables.

Finally, Galan-Gonzalez *et al.* (14) found a link between breastfeeding and the presence of diastema in temporary dentition, however we could not confirm any association between breastfeeding and diastema in mixed dentition.

As regards the association between the duration of breastfeeding and the need for orthodontic treatment determined by IOTN and DAI, there is no data in the scientific literature to compare with. No association was found between duration of breastfeeding and orthodontic treatment need determined by the DAI. In the case of the treatment need determined by the IOTN we obtained a 0.056 P value in the Chi-square test which, even if it is not significant, comes very close to the statistical significance.

This work presents as its main innovation the study of the association between breastfeeding and orthodontic treatment need at the age of 9 as determined by internationally recognised indexes: the IOTN and DAI, as no previously published study analysing such an association has been found. It also analyses the association between breastfeeding and numerous occlusal traits (overjet, overbite, openbite, midline displacement, incisor crowding, incisor spacing, diastema, maximum maxillary and mandibular irregularity, anterior crossbite and posterior crossbite), in contrast to the majority of previous studies which investigate the association between breastfeeding and a small selection of occlusal traits (9-18,28,29). The distribution of our groups factored in the current duration of maternity leave in Spain (16 weeks).

Furthermore, the majority of publications analysing the association between breastfeeding and occlusal traits are based on studies using younger samples at the primary dentition stage. Whilst in mixed dentition, as in the present study, only four studies focused on breastfeeding and a few occlusal traits (9,28,29).

It is true that the retrospective nature of our study might suggest a bias in the breastfeeding data collection. However, we must highlight that the breastfeeding information was recorded in medical histories and collected directly from the mothers during the infant’s first two years of life. Furthermore, we can suggest that given the favourable conditions of the dental examination and examiner’s calibration, our data could be used as a reference in future research in which it might be possible to define the confounder effect of the presence of some oral habits.

We can conclude that breastfeeding has a protective effect against posterior crossbite that extends through into the mixed dentition stage, with a decreased prevalence of posterior crossbite as the duration of breastfeeding rises. Similar effects against the rest of the examined occlusal traits were not found.

No statistically significant association has been observed between the duration of breastfeeding and orthodontic treatment need at age 9 as determined by the DAI and IOTN, although the association between the duration of breastfeeding and orthodontic treatment need, determined by the IOTN, is found to be very close to the statistical significance (*p*=0.056).
